# PARP inhibitors elicit distinct transcriptional programs in homologous recombination competent castration‐resistant prostate cancer

**DOI:** 10.1002/1878-0261.70098

**Published:** 2025-09-07

**Authors:** Moriah L. Cunningham, Jasibel Vasquez‐Gonzalez, Samantha M. Barnada, Salome Tchotorlishvili, Latese Jones, Ryan Maguire, Genevieve Lewis, Kinza Rizwan, Jenny Deng, Salma Koachar, Drithi Patel, Hailey Shankle, Tessa Mulders, Namra Ajmal, Charalambos Solomides, Emad S. Alnemri, Teresa F. Alnemri, Ayesha A. Shafi, Leonard G. Gomella, Wm Kevin Kelly, Steven B. McMahon, Matthew J. Schiewer

**Affiliations:** ^1^ Department of Cell Biology and Regenerative Medicine Thomas Jefferson University Philadelphia PA USA; ^2^ Department of Biochemistry and Molecular Biology, Sidney Kimmel Medical College Thomas Jefferson University Philadelphia PA USA; ^3^ Department of Pharmacology, Physiology, and Cancer Biology Thomas Jefferson University Philadelphia PA USA; ^4^ Department of Medicine, Section Hematology/Oncology Baylor College of Medicine Houston TX USA; ^5^ Department of Pathology and Genomic Medicine Thomas Jefferson University Hospital Philadelphia PA USA; ^6^ Center for Prostate Disease Research, Murtha Cancer Center Research Program, Department of Surgery Uniformed Services University of the Health Sciences Bethesda MD USA; ^7^ The Henry M. Jackson Foundation for the Advancement of Military Medicine, Inc. Bethesda MD USA; ^8^ Sidney Kimmel Comprehensive Cancer Center Thomas Jefferson University Philadelphia PA USA; ^9^ Department of Urology, Sidney Kimmel Cancer Center Thomas Jefferson University Philadelphia PA USA; ^10^ Department of Medical Oncology, The Sidney Kimmel Comprehensive Cancer Center Thomas Jefferson University Philadelphia PA USA; ^11^ Department of Urology Thomas Jefferson University Philadelphia PA USA

**Keywords:** p53, PARP, PARP inhibitors, PEITC, prostate cancer, racial disparities

## Abstract

Prostate cancer (PCa) is the second most lethal cancer in men in the US. African American (AA) men have twice the incidence and death rate of European American (EA) men. Advanced PCa shows increased expression and activity of the DNA damage/repair pathway enzyme, poly (ADP‐ribose) polymerase 1 (PARP1). PARP1 inhibitors (PARPi) are FDA‐approved for advanced PCa tumors with mutations in the homologous recombination repair (HRR) pathway. However, PARPi can provide benefit in model systems without HRR deficiencies. PARPi have distinct biochemical mechanisms, potencies, and toxicity profiles. While there is emerging evidence of differences in DNA damage/repair pathway enzyme expression between EA and AA men, PARP1 expression has not been fully explored in the context of race. This study hypothesized: (a) AA and EA PCa may respond differently to PARPi and (b) different PARPi may uniquely impact the transcriptome, irrespective of HRR status. Study results indicate a link between racial background and PARP1 expression/activity and define unique and overlapping transcriptional responses downstream of all five PARPi. These findings may lead to refined personalized recommendations for use of specific PARPi.

## Graphical abstract

PARP inhibitors are used to treat a small subset of prostate cancer patients. These studies reveal that PARP1 activity and expression are different between European American and African American prostate cancer tissue samples. Additionally, different PARP inhibitors cause unique and overlapping transcriptional changes, notably, p53 pathway upregulation. These studies can ultimately lead to advances in personalized PARP inhibitor treatment options.

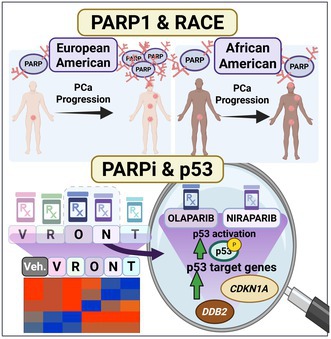

**Abbreviations**
AA: African American
ADPHPD: adenosine diphosphate‐hexaphosphate dehydrogenase
ADT: androgen deprivation therapy
AR: androgen receptor
BRCA1/2: breast cancer gene 1 and 2
CDT: Charcoal dextran treatment
CRPC: Castration‐resistant prostate cancer
CVCL: Cellosaurus cell line identification code
DDB2: damage‐specific DNA binding protein 2
DEG: differentially expressed gene
DMEM: Dulbecco's modified Eagle medium
DMSO: dimethyl sulfoxide
DNA: deoxyribonucleic acid
EA: European American
FBS: Fetal bovine serum
FDA: U.S. Food and Drug Administration
HRR: homologous recombination repair
IMEM: improved minimum essential medium
IRB: Institutional Review Board
KEGG: Kyoto Encyclopedia of Genes and Genomes
MTT: 3‐(4,5Dimethylthiazol2yl)2,5diphenyltetrazolium bromide
NAD^+^
: nicotinamide adenine dinucleotide
PAR: poly(ADP‐ribose)
PARP1: poly(ADP‐ribose) polymerase 1
PARPi: poly(ADP‐ribose) polymerase inhibitor
PBS: phosphate‐buffered saline
PEITC: phenethyl isothiocyanate
PMSF: phenylmethylsulfonyl fluoride
PVDF: polyvinylidene difluoride
qPCR: quantitative polymerase chain reaction
RIPA: radioimmunoprecipitation assay
RNA: ribonucleic acid
RPLP0: ribosomal protein lateral stalk subunit P0
RPMI 1640: Roswell Park Memorial Institute 1640 medium
RPS4X: ribosomal protein S4, X‐linked
RRID: research resource identifier
RT: room temperature
SKCCC: Sidney Kimmel Comprehensive Cancer Center
SU2C: stand up to cancer
TCGA: The Cancer Genome Atlas
TMA: tissue microarray
TNMplot: tumor–normal matched plot
TP53: tumor protein P53
TSA: trichostatin A
UQCRQ: ubiquinol‐cytochrome C reductase, complex III, subunit VII
US: United States
WT: wild‐type

## 1 Introduction

Prostate cancer (PCa) is the most commonly diagnosed malignancy in American men, where it accounts for 29% of all cases [1]. PCa is the second leading cause of cancer‐related death in American men [1, 2]. The disease disproportionately affects African American (AA) men. AA men bear a burden twice as high as EA men for developing and dying from PCa [2]. Despite these trends, research exploring these race‐based differences remains limited [3]. Targeting DNA damage repair pathways has become a viable strategy for cancer treatment [4], and recent findings suggest that there are differences between AA and EA DNA damage response pathways that could be therapeutically exploited [5, 6, 7]. Collectively, these studies suggest that AA men with PCa have higher DNA damage/repair enzyme mutations and lower overall DNA damage/repair activity than EA men. These biological differences can impact AA men's responsiveness to DNA damage/repair therapies.

Poly(ADP‐ribose) polymerase 1 (PARP1) is a central component of the cellular DNA damage and repair pathway, and PARP inhibitors (PARPi) are currently used in the management of a subset of prostate, breast, ovarian, and pancreatic cancers [8, 9, 10, 11, 12]. PARPi block PARP1 enzymatic activity (PARylation) [13, 14]. PARylation is elevated as a function of PCa disease progression [8]. While the PARP family of enzymes is large, the major target of PARPi is PARP1 [15, 16, 17]. The best‐characterized role for PARP1 is in the recognition of single‐stranded DNA breaks at replication forks and elsewhere in the genome [18]. Upon recognition of these breaks, PARP1 becomes PARylated. PARylation is marked by NAD^+^ acting as a co‐factor to attach ADP‐ribose groups to PARP1 to generate PAR chains [14]. PARP1‐induced PARylation at these sites of damage triggers recruitment of additional repair proteins [18]. PARPi block PARP1 function by either inhibiting its initial recruitment to DNA damage sites or by trapping the enzyme at recruitment sites [19].

PARPi treatment becomes lethal in cancer cells harboring defects in homologous recombination‐based repair (HRR) pathways, such as those resulting from mutation of *BRCA1/2* [20]. However, it is now clear that some HRR‐competent tumors can respond to PARPi, and that not all HRR‐defective tumors are PARPi responsive [21, 22]. Clinically, patients who receive the most benefit from PARPi have HRR incompetencies [22]. Nonetheless, recent clinical trials show the benefit of PARPi in PCa patients, regardless of HRR status (NCT03748641, NCT03732820, NCT03395197) [23, 24]. The clinical PARPi, Olaparib, Rucaparib, Niraparib, and Talazoparib are currently approved in HRR‐defective PCa, primarily for use as a combination therapy with androgen receptor (AR)‐directed therapy [24, 25, 26, 27, 28, 29]. Veliparib is not approved in PCa as a single agent or a combination therapy currently, although it is actively used in clinical trials for the disease [30, 31].

PARP1 has nuclear functions outside of the canonical DNA damage response, including a role in transcriptional regulation [3]. For example, PARP1 regulates key oncogenic transcription factors involved in PCa progression, such as AR and E2F1 [8, 32]. Recent clinical trials in PCa have demonstrated that PARPi in combination with AR‐directed therapy is superior to either modality alone [33, 34, 35]. Despite the increased sensitivity, a meta‐analysis indicates a differential benefit depending on the specific PARPi utilized and HRR biomarker status [36]. Given the critical role PARP1 activity plays in transcriptional regulation and the varied clinical trial data, it was hypothesized that the different PARPi elicit distinct transcriptional programs that have biological relevance in the management of PCa. Additionally, given the differences in efficacy and toxicity between the different PARPi, understanding distinctions in the activation of downstream pathways is likely to help refine the choice of inhibitor that depends on the integrity of these pathways in a given patient.

The studies herein reveal a difference in the expression of PARP1 and PARylation between EA and AA PCa tumor samples. They also reveal that clinical PARPi elicit both overlapping and distinct gene expression programs across several HRR‐competent PCa model systems. PARPi demonstrate highly variable dose responses, and when combined with androgen deprivation therapy (ADT), all PARPi tested are more effective at limiting PCa cell growth in *in vitro* model systems. Moreover, distinct PARPi differentially impact the p53 pathway. Collectively, these studies identify that PARPi elicit distinct transcriptional programs that may impact the response to PARPi in HRR‐competent PCa.

## 2 Materials and methods

### 2.1 Tissue microarray (TMA)

Briefly, the Sidney Kimmel Comprehensive Cancer Center (SKCCC) TMA was generated from prostate biopsies with a total of 94 PAR samples and 95 PARP1 samples. TMAs were assigned a Gleason number. Ninety‐four samples were scored by a pathologist for PAR; 95 samples were scored by a pathologist for PARP1. Patient information was stratified via Gleason number and age at diagnosis (Tables 1 and 2). Both Gleason and PAR and PARP1 scores were given by a board‐certified pathologist. No normal or non‐neoplastic specimens were within this dataset. Because these TMAs were obtained from the SKCCC biorepository, they are IRB exempt.

**Table 1:** Patient information for PARP1 stained tissue samples. Data are presented as *n* (%). Percentages were rounded to the nearest whole number.

	European American (*n* = 19)	Black or African American (*n* = 76)
Age at diagnosis < 50	3 (16%)	14 (18%)
Age at diagnosis > 50	16 (84%)	62 (84%)
Gleason number		
3 + 3	9 (47%)	38 (50%)
3 + 4	5 (26%)	27 (35%)
4 + 3	5 (26%)	10 (13%)
4 + 4	0 (0%)	1 (5%)

**Table 2:** Patient information for PAR‐stained tissue samples. Data are presented as *n* (%). Percentages were rounded to the nearest whole number.

	European American (*n* = 18)	Black or African American (*n* = 76)
Age at diagnosis < 50	3 (17%)	14 (18%)
Age at diagnosis > 50	15 (83%)	62 (82%)
Gleason number		
3 + 3	8 (44%)	39 (51%)
3 + 4	5 (28%)	27 (36%)
4 + 3	5 (28%)	10 (13%)
4 + 4	0 (0%)	0 (0%)

### 2.2 Cell lines, cell culture, and treatment conditions

The PCa cell line, MDA‐PCa‐2b (RRID: CVCL_4748), was generously shared by the late Dr Nora Navone at the University of Texas MD Anderson Cancer Center [37]. MDA‐PCa‐2b use for experiments is in culture within 1 month with less than five passages after thawing out. Cells were maintained in HPC1 (Fisher Cat# BRFF‐HPC1; Waltham, MA, USA) supplemented with 20% FBS (HI) and Pen‐Strep (50 units·mL^−1^–50 mg·mL^−1^ final concentration) in culture vessels pre‐coated with ATHENA ES FNC COATING MIX HUMAN (Fisher Scientific #NC2000952). Mycoplasma tests were performed routinely, every 2 weeks, while cells were in culture with PCR Mycoplasma Detection Kit (Alfa Aesar #J66117‐&J; Ward Hill, MA, USA) and STR Fingerprinting validation was performed by MD Anderson Cancer Center Cytogenetics and Cell Authentication Core. The PCa cell lines C4‐2 (RRID:CVCL_GY03), LNCaP (RRID:CVCL_0395), DU145 (RRID:CVCL_0105), and 22RV1 (RRID:CVCL_1045) were purchased from ATCC. ATCC performs cell authentication processes as part of their quality control prior to shipment. More information on their quality commitment can be found on their website: https://www.atcc.org/about‐us/quality‐commitment. Cells were not cultured for longer than 6 months after receipt from their original source, or no longer than 45 passages. Cells were routinely mycoplasma tested every 6 months. C4‐2 and LNCaP were maintained in minimum essential media (IMEM) supplemented with 5% heat activated fetal bovine serum (FBS) with 1% l‐glutamine (100 units·mL^−1^) and 1% penicillin–streptomycin (100 units·mL^−1^) or RPMI 1640 supplemented with 10% heat inactivated fetal bovine serum (FBS) with 1% l‐glutamine (100 units·mL^−1^) and 1% penicillin–streptomycin (100 units·mL^−1^). DU145 and 22RV1 were maintained in Dulbecco's modified Eagle medium (DMEM) with 10% FBS and 1% l‐glutamine and 1% penicillin–streptomycin. The cells were all cultured at 37 °C in an atmosphere of 5% CO_2_. Cells were treated as indicated with the PARP inhibitors (PARPi) Olaparib (Selleck S1060; Houston, TX, USA), Talazoparib (Selleck S7048), Rucaparib (Selleck S4948), Niraparib (Selleck S2741), or Veliparib (Selleck S1004). All PARPi were kept as 100 mm stock aliquots and stored at −80 °C until use.

### 2.3 Proliferation analyses

#### 2.3.1 QuantFlor ONE dsDNA

Cells were seeded in FBS‐supplemented media in 96‐well plates to reach 80% confluence. The cells were treated with a vehicle control (DMSO) or a PARPi (Veliparib, Niraparib, Olaparib, Talazoparib, or Rucaparib) at varying doses from 0.001 to 150 μm. At 96 h, the media was discarded from the plates, and the plates were gently washed with PBS. After washing, 50 μL of ddH_2_O was added to each well and placed in a 37 °C incubator for 1 h. 50 μL of QuantFuour dsDNA dye system (Promega E2670; Madison, WI, USA) was added to the cells and incubated for 5 min at room temperature (RT) and protected from light exposure. A Promega GloMax Discover plate reader was utilized to quantify the fluorescent signal. The results were normalized to the vehicle control to assess cell viability. These experiments were conducted in each cell line in technical triplicate and in three independent experiments.

#### 2.3.2 Crystal violet

Cells were seeded to reach 80% confluency within 96 h in a 6‐well plate. At 96 h, the media was discarded, and the cells were formalin fixed (1 mL formalin per well) at room temperature for 15 min. Formalin was discarded, and 0.5% Crystal Violet solution was added to the well at 1 mL each for an additional 15 min. The Crystal Violet solution was decanted from the cells with careful submersion in room temperature water. Plates were then dried overnight and scanned with EPSON V600 to obtain qualitative analysis of cell growth. For quantitative analysis, the dye was eluted at RT in Sorensen Buffer. Optical density was used to analyze the eluted dye using the Promega GloMax Discover plate reader. Results were normalized to DMSO.

#### 2.3.3 MTT

MTT assays were conducted to assess cell viability following treatment with four different PARP inhibitors. All experiments were performed in 24‐well plates, with each treatment condition tested in triplicate. MDA‐PCa‐2b cells were seeded at a density of 3 × 10^4^ cells per well in 500 μL of BRFF‐HPC‐1 complete serum‐free medium (Cat. No. NC9970798), supplemented with 20% fetal bovine serum (FBS; Cat. No. 35‐075‐CV) and 50 μg·mL^−1^ Penicillin–Streptomycin (Pen‐Strep; Cat. No. 15140122). Plates were incubated at 37 °C in a humidified incubator with 5% CO₂, and cells were allowed to attach for 24 h prior to treatment. Next, cells were treated with fresh medium containing increasing concentrations of the following PARP inhibitors: Veliparib (ABT‐888) (Cat. No. S1004, Batch No. 17), Rucaparib (AG‐014699) (Cat. No. S1098, Batch No. 13), Olaparib (AZD2281) (Cat. No. S1060, Batch No. 23), or Niraparib (MK‐4827) (Cat. No. S2741, Batch No. 05). Appropriate vehicle controls (0.1% DMSO, ethanol, or nuclease‐free water) were included for each drug. After 96 h of treatment, 50 μL of a 10× MTT stock solution (5 mg·mL^−1^ in PBS) was added to each well (final concentration: 0.5 mg·mL^−1^). Plates were incubated for an additional 2 h at 37 °C. Following incubation, 750 μL of solubilization solution (1 : 1 v/v HCl : isopropanol) was added to each well. Plates were gently shaken on an orbital shaker for 25 min at room temperature in the dark to allow complete dissolution of the formazan crystals. Absorbance was measured at 570 nm with background subtraction at 630 nm using a BioTek Cytation 5 microplate reader. Background‐corrected absorbance values (OD570–OD630) were normalized to the vehicle control group and expressed as percent viability relative to control. Data were analyzed and plotted using graphpad prism software (LLC, San Diego, CA, USA), and dose–response curves were generated to determine the half‐maximal inhibitory concentration (IC₅₀) for each PARP inhibitor. IC₅₀ values were calculated using nonlinear regression analysis (log[inhibitor] vs. response–variable slope). All results are presented as the mean ± standard deviation (SD) of triplicate wells.

#### 2.3.4 Incucyte

C4‐2 cells were seeded to reach 80% confluence at 96 h on 24‐well plates. They were seeded in FBS growth conditions or charcoal dextran stripped (CDT) media conditions. C4‐2 seeded in FBS were seeded at 50 000 cells per well on 24‐well plates and 150 000 cells per well on 6‐well plates. C4‐2 seeded in CDT were seeded at 75 000 cells per well on 24‐well plates. Cells were treated with either DMSO or a PARPi at their IC50 value. After treatment, they were put into the Incucyte to quantify cell growth over a 96 h time series.

#### 2.3.5 Synergy experiments

DU145 cells were seeded to reach 80% confluency in 96‐well plates. Cells were treated with vehicle control (DMSO), PEITC (HY‐23155), or Olaparib. PEITC and Olaparib were administered alone or in unique combinations of both agents. 96 h posttreatment, QuantiFlor ONE dsDNA results were quantified on a Promega Glomax Plate reader. Results were analyzed in ComBenefit as described by the original authors [38]. All graphical representations of synergy were produced by the combenefit software (https://sourceforge.net/projects/combenefit/).

### 2.4 RNA sequencing and analysis

C4‐2 cells were steroid depleted for 72 h and then treated with a PARP inhibitor (Veliparib, Rucaparib, Olaparib, Niraparib, or Talazoparib) or vehicle control (DMSO) in triplicate. RNA was extracted and purified using Trizol reagent per the manufacturer's instructions. Novogene (Sacramento, CA, USA) performed the library preparation and next‐generation sequencing following their company policies. Briefly, mRNA was purified using poly‐T magnetic beads, fragmented, and a directional library was prepared for sequencing on an Illumina platform. FastQC (https://github.com/s‐andrews/FastQC) was used for quality control of all raw fastq files and adapters were removed using TrimGalore! (https://github.com/FelixKrueger/TrimGalore). Reads mapping to each gene in each sample were quantified to abundance using kallisto [39]. Abundance was then converted to raw gene counts via deseq2 [40]. The raw gene count for each sample was used to determine differential gene expression (*P*‐value < 0.05; FDR < 5%) and were grouped based on PARP inhibitor treatment (Veliparib, Rucaparib, Olaparib, Niraparib, or Talazoparib) or control (DMSO). All statistical analyses were performed using kallisto v0.50.0, deseq2 v1.40.2, and r v4.2.3. Reactome pathway analysis was performed via webgestalt from the differential gene expression list as determined by deseq2.

### 2.5 qPCR validation

C4‐2, LNCaP, DU145, and 22RV1 were seeded to reach 80% confluence in FBS. C4‐2 cells were also seeded to reach 80% confluence in CDT and allowed 72 h of steroid depletion before vehicle control or PARPi treatment. Cells were harvested in trypsin, and RNA was isolated using Trizol based on the manufacturer's instructions.

#### 2.5.1 Primers

The following primers were used for quantitative PCR experiments: RPLP0 (fwd: CAGCATCTACAACCCTGAAG, rev: GACAGACACTGGCAACATT), TP53 (fwd: CCCAAGCAATGGATGATTTGA, rev: GGCATTCTGGGAGCTTCATCT), CDKN1A (fwd: GGCAGACCAGCATGACAGATT, rev: GCGGATTAGGGCTTCCTCT), and DDB2 (fwd: GCTGAACATGGACGGCAAAG, rev: CCATCGGGACTGAAACAAGC).

### 2.6 Immunoblotting

For regular growth conditions, cells were seeded and treated with vehicle control or one of the five PARPi at the IC50 value on a 10‐cm plate. For steroid‐depleted growth conditions, cells were seeded, steroid‐depleted for 72 h, and then treated with varying PARPi for 24 h. Immunoblots were quantified using imagej(Madison, WI, USA) software and normalized to the vehicle control.

C4‐2, LNCaP, DU145, and 22RV1 were seeded to reach 80% confluence in FBS. C4‐2 cells were also seeded to reach 80% confluence in CDT and allowed 72 h of steroid depletion before vehicle control or PARPi treatment. All cells were harvested and lysed for protein analysis. Whole‐cell lysates were prepared using RIPA buffer containing 100× ADPHPD (adenosine diphosphate (hydroxymethyl) pyrrolidinediol) Millipore (Burlington, MA, USA), D43216, 1 m NaF, 100× protease inhibitor, 100× PMSF, 13 mg B‐glycerophosphate, and 13 mg sodium orthovanadate, or 100× protease inhibitor and TSA. Thermo Scientific NanoDrop OneC Protein Lowry Assay was used to quantify protein concentration. 30 μg of protein were loaded on a 10% Tris‐Glycine gel (Bio‐Rad, Hercules, CA, USA) and transferred onto a PVDF membrane. Membranes were blocked overnight in 5% dry milk at 4 °C. Membranes were then blocked overnight in primary antibody.

#### 2.6.1 Antibodies

The following antibodies used for immunoblots are listed as ‘antibody (antibody type, commercial supplier, catalog #)’: PARP1 N‐terminal Antibody (polyclonal, Active Motif, 39559), PAR Anti‐poly‐ADP‐ribose binding reagent (polyclonal, Millipore Sigma, MABE1031), B‐actin (monoclonal, Santa Cruz, sc‐47778; Santa Cruz, CA, USA), B‐actin (recombinant monoclonal, Cell Signaling, mAb #4970; Danvers, MA, USA), p53 (monoclonal, Santa Cruz, sc‐126), p21 (polyclonal, ABCAM, ab227443; Cambridge, UK) Vinculin (monoclonal, Sigma‐Aldrich, V9264; St. Louis, MO, USA).

### 2.7 Statistical analysis

All experiments were performed in technical triplicate with at least three biological replicates per condition. Statistical significance was determined by using Student's *t*‐test or one‐way ANOVA on graphpad prism as appropriate. All data were analyzed using Microsoft Excel, prism graphpad, and imagej.

## 3 Results

### 3.1 PARP1 and PAR immunoreactivity in a racially diverse PCa patient cohort

**Fig. 1:**
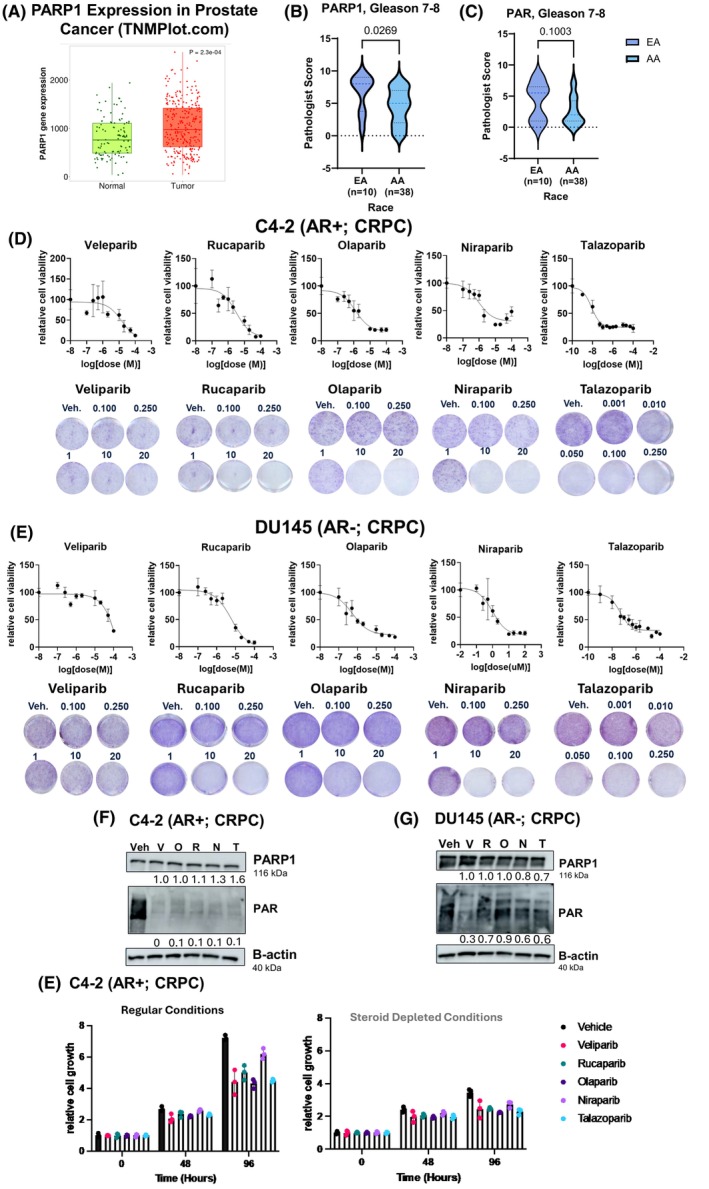
Clinical PARPi elicit distinct antitumor activity in HR‐competent, PCa model systems. (A) TNMplot.com expression of *PARP1* between normal prostate (*n* = 106) and tumoral prostate (*n* = 283) tissue (*P* = 2.3e‐04). (B, C) PARP1 (B) and PAR (C) scores for Gleason 7–8 PCa TMA samples in EA (*n* = 10) vs. AA (*n* = 38) patients. AA, African American; EA, European American. (D, E) PARPi (Veliparib, Rucaparib, Olaparib, Niraparib, or Talazoparib) IC50's in C4‐2 (AR^+^, CRPC) (D) and DU145 (AR^−^, CRPC). CRPC, Castrate Resistant Prostate Cancer. Cells were treated vehicle control or a PARPi for 72 h. dsDNA Quantiflor One assay was performed to generate IC50 curves. Crystal violet validation of PARPi IC50 underneath each graph and in Fig. S2. Error bars represent standard deviation (SD) (D, E) from three, independent experiments. (F, G) Immunoblots of PARP1 and PAR levels after treatment with PARPi IC50 values found in C4‐2 (F) and DU145 (G). Normalized to b‐Actin. N, Niraparib; O, Olaparib; R, Rucaparib; T, Talazoparib; V, Veliparib. Representative of three, independent replicates in C4‐2 (*n* = 3) and DU145 (*n* = 3). (H) C4‐2 treated with regular growth conditions (left) or steroid depletion conditions (right) ± PARPi. Time points of 0, 48 and 96 h were analyzed for cell growth with the Incucyte. Error bars represent the standard error of the mean (SEM) between four, independent experiments. Each experiment had three biological replicates per experiment.

In order to broaden our understanding of the link between PARP1 mRNA expression and Pca disease progression, *PARP1* expression was queried using the publicly available web‐based tool, TNMplot [41]. This analysis revealed that *PARP1* expression in prostate tumor samples is significantly higher than in normal prostate samples (Fig. 1A). However, there is no publicly available race‐related data associated with these cohorts. Therefore, a racially diverse tissue microarray (TMA) cohort was obtained from the Sidney Kimmel Comprehensive Cancer Center (SKCCC) to investigate the impact of race stratification on PARP1 protein and PAR levels in Pca disease progression (Fig. 1B,C and Fig. S1A–D). The cohort is approximately 80% AA (76 samples) and 20% EA (18 samples) (Tables 1 and 2). Race‐based information was self‐reported. The TMA samples were analyzed by a board‐certified pathologist and assigned a Gleason number. Samples with Gleason 6 were classified as early‐stage Pca, whereas samples with Gleason 7–8 were classified as advanced Pca. The samples were subsequently immunohistochemically stained for PARP1 protein expression and activity (PAR). In the overall cohort, PARP1 and PAR expression increase as a function of disease progression, similar to previously published data (Fig. S1A) [42, 43, 44]. When stratified by race, however, EA samples have higher overall PARP1 and PAR expression than AA samples, irrespective of disease progression (Fig. 1B,C and Fig. S1B). In the EA samples, PARP1 and PAR expression increase substantively as a function of disease progression (Fig. 1B,C, left and Fig. S1B, left). In contrast, in AA samples, PARP1 and PAR do not increase as a function of disease progression (Fig. S1C, right and Fig. S1D, right). Collectively, these data indicate that PARP1 protein and PARylation expression are higher in EA tumor samples than AA samples and increase substantively between disease states exclusively in EA samples within this cohort. These data raise the possibility that EA may be more responsive in general to PARP inhibition due to increased PARylation correlated with more progressive disease within this cohort. These data also raise the possibility that AA men may be less responsive to PARP inhibition due to PARylation not increasing as a function of disease progression within this cohort. This preliminary observation alone suggests that detailed preclinical and clinical studies are warranted to further assess the impact of PARPi in Pca patients of different Gleason numbers and different racial backgrounds.

### 3.2 Clinically relevant PARPi elicit distinct effects on viability in HRR‐competent Pca model systems

Since PARP1 activity increased with disease severity in the EA TMA samples and not the AA TMA samples, it was hypothesized that the AA‐derived Pca cell line, MDA‐Pca‐2B, may be less responsive to PARPi treatment *in vitro*. This was hypothesized because a previous study assessing increased PAR activity in Pca patient samples rationalized the potential responsiveness of Pca to PARPi [8].

After treatment with one of four clinical PARPi (Veliparib, Rucaparib, Olaparib or Niraparib), IC50 values were determined in MDA‐Pca‐2B with a colorimetric‐based assay. No biologically relevant doses were obtained in this model system, with IC50 values in the 20–400 μm range (Fig. S1E). The lack of responsiveness of AA cell lines to PARPi coupled with the TMA results caused studies in EA Pca cell lines to be prioritized for further analysis. Furthermore, understanding the mechanism of PARPi in HRR‐competent models is far more limited than the understanding in HRR‐defective settings. To better understand PARPi response in HRR‐competent, EA‐derived Pca cell lines, C4‐2, DU145, LNCaP, and 22RV1 cells were treated with one of the five clinical PARPi (Veliparib, Rucaparib, Olaparib, Niraparib, or Talazoparib) (Fig. 1D–H and Figs S2 and S3). In all cell lines, PARPi efficacy patterns were consistent with previous studies, where Veliparib has the highest IC50 and Talazoparib the lowest IC50 (Figs S2 and S3A–E).

In general, C4‐2, a castration‐resistant prostate cancer (CRPC) HRR‐competent cell line, was the most sensitive to PARPi treatment (Fig. 1D,F). In marked contrast, DU145, a cell line that represents more aggressive CRPC, was the least sensitive to PARPi treatment, with one of the highest IC50 values within the EA cell model system (Fig. 1E,G). C‐42 and DU145 responded similarly to Olaparib and Niraparib treatments, despite DU145 generally being more aggressive and less responsive to Pca treatment options than the other cell lines. For all other PARPi tested, C‐42 was more sensitive to treatment than DU145. LNCaP, a hormone therapy‐sensitive cell line, and 22RV1, another CRPC model, were also assessed (Figs S2 and S3). The IC50 values for all PARPi in all cell lines were validated by crystal violet staining, with the results following the same general trend found using the fluorescence‐based assay (Fig. S2). The IC50 values from the fluorescence‐based assay were used for future analyses due to the smaller standard deviation between experiments and more precise readouts. The effects of the PARPi IC50 values on PARP1 expression and PARylation were assessed. At the IC50 value for each cell line, all five PARPi decrease PARP1 enzymatic activity (PARylation) without impacting PARP1 protein expression, as expected (Fig. 1F,G and Fig. S3A–D).

Clinically, patients receiving PARPi must have an HRR defect in genes such as *BRCA1* or *BRCA2* based on FDA approval criteria [45]. However, previous studies and recent clinical trials indicate that PARPi can elicit antitumor responses in non‐HRR‐defective contexts [8, 46, 47, 48]. Given that C4‐2 is an HRR‐competent model that is more responsive to PARPi than the other cell lines assessed above, the impact of *BRCA2* manipulation on PARPi response was measured. Talazoparib, the PARPi that decreased cell growth the most and had the highest potency in the C4‐2 model, was used for this analysis (Fig. 1D,H). Transient depletion of *BRCA2* using si‐RNA elicited a more pronounced decrease in cell growth when compared to Talazoparib alone, as expected (Fig. S3F). These results confirm that the C4‐2 cell model recapitulates the well‐defined synergistic effects of *BRCA2* impairment and PARPi shown in other models [49, 50].

Advanced Pca patients typically receive ADT prior to and in combination with other treatment options, including PARPi [33, 35, 51]. Recent clinical trials indicate a benefit from combining PARPi with ADT treatment in CRPC patients [52]. In the current study, PARPi response in the presence of steroid depletion was assessed in the C4‐2 (CRPC) model, again based on the relatively high sensitivity to PARPi. Upon PARPi treatment in normal growth conditions, all PARPi elicited an anti‐proliferative effect compared to vehicle control (Fig. 1H). Similarly, in the presence of steroid depletion, PARPi elicits an anti‐proliferative effect compared to vehicle control (Fig. 1H). These observations demonstrate that clinical PARPi can differentially decrease cell viability in HRR‐competent Pca model systems, even in the presence of steroid deprivation.

### 3.3 Clinically relevant PARPi elicit both overlapping and distinct changes in gene expression

**Fig. 2:**
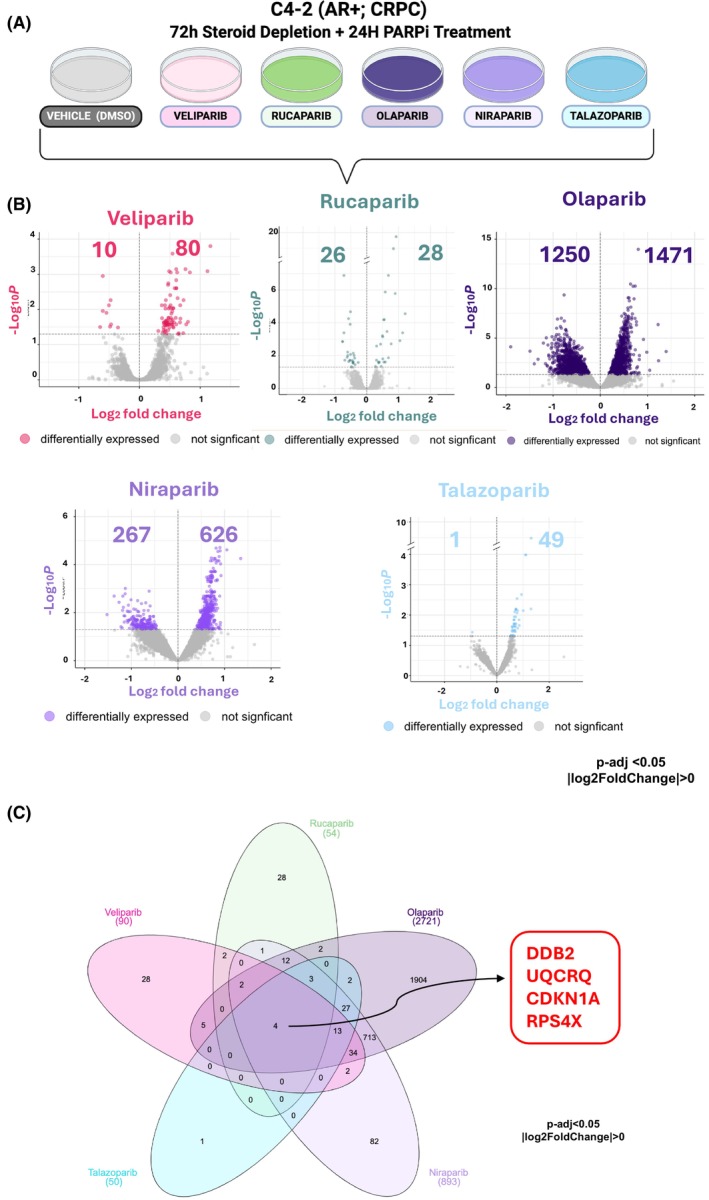
Clinical PARPi elicit both overlapping and distinct alterations in gene expression. (A) Experimental design for treatment with PARPi. C4‐2 cells were steroid depleted for 72 h before introducing either vehicle control (DMSO) or a PARPi (Veliparib, Rucaparib, Olaparib, Niraparib, Talazoparib) to the cells. PARPi, PARP1 inhibitor. RNA sequencing was performed on each condition (with Novogene). Experiments are the result of four independent experiments. (B) Volcano plots of differentially expressed genes in each PARPi treatment condition relative to vehicle control. (C) Lotus plot of the number of DEGs in each PARPi treatment condition relative to vehicle control.

PARP1 has a role in the transcriptional regulation of key Pca oncogenes, which presumably explains aspects of PARPi efficacy [8, 32]. No studies to date have compared the impact of all five PARPi on transcriptional activity in an HRR‐competent context. In the C4‐2 model, this study found a vast range of IC50 values between PARPi (14.44 μm to 775 nm). Previous studies reported differential efficacies among the five clinical PARPi used in Pca [23, 24, 36]. Combining these differences in IC50 values with the different clinical benefits led to the hypothesis that the clinically relevant PARPi may be differentially impacting the transcriptome and thereby activating distinct downstream pathways. Therefore, the transcriptional changes in response to all five PARPi in the HRR‐competent CRPC model C4‐2 were assessed. To accomplish this, cells were steroid depleted for 72 h followed by treatment with a vehicle control or one of the clinical PARPi at the IC50 doses determined in Figs 1 and 2A. RNA sequencing was performed on C4‐2 cells following treatment to visualize the impact of the individual PARPi on gene expression (Fig. 2A and Figs S4 and S5, Table S1). Treatment with Veliparib, Rucaparib, Olaparib, Niraparib, and Talazoparib resulted in the downregulation of 10, 26, 1250, 267, and 1 gene respectively, and the upregulation of 80, 28, 1471, 626, and 49 genes respectively (Fig. 2B). All five PARPi impacted key KEGG and Hallmark pathways that have previously been associated with response to PARPi, including cell cycle, DNA repair, and cell death (Fig. S5). Despite these similarities, each PARPi also displayed differential effects on the transcriptome of HRR‐competent CRPC cells.

To discern the impact on gene expression and streamline the pathways for further investigation, the genes affected by each distinct PARPi were compared to one another in a lotus plot. Olaparib treatment impacted a broad set of distinct genes (*n* = 1904), whereas Talazoparib treatment had the least impact on distinct gene expression (*n* = 1) (Fig. 2C). Veliparib, Rucaparib, and Niraparib treatment resulted in the unique changes of 28, 28, and 82 genes, respectively (Fig. 2C). All other genes impacted by PARPi treatment overlapped with at least one other PARPi. Four genes were impacted across all PARPi treatments: *DDB2*, *UQCRQ*, *CDKN1A*, and *RPS4X* (Fig. 2C). These findings demonstrate that despite similarities between the PARPi, individual PARPi can differentially impact the transcriptome of Pca, which may be associated with the differential antitumor effectiveness of the PARPi tested in this study.

### 3.4 p53‐related pathways are enriched among genes impacted by Olaparib and niraparib treatment

**Fig. 3:**
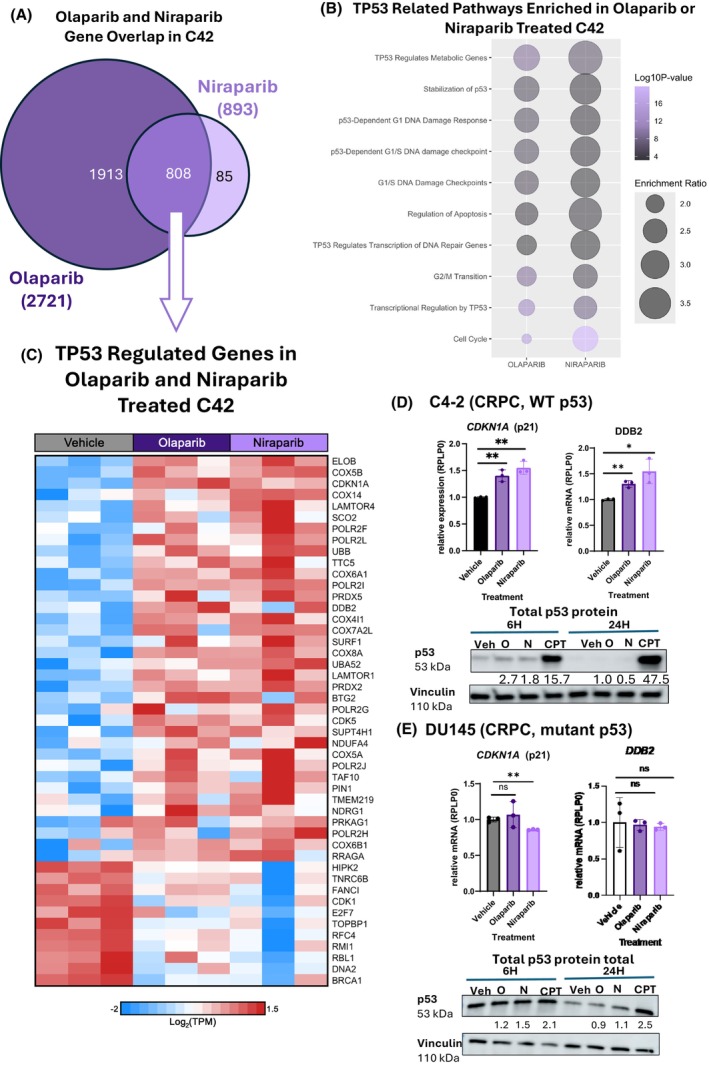
p53‐related pathways are enriched in response to Olaparib and Niraparib treatment in a p53 WT model system. (A) Venn Diagram of the number of differentially expressed genes after Olaparib or Niraparib treatment. (B) Dot plot of TP53‐related Reactome pathways enriched in Olaparib‐ or Niraparib‐treated C4‐2. (C) Heatmap of TP53‐regulated genes in Olaparib‐ and Niraparib‐treated C4‐2 compared to vehicle control. (D, E) Relative expression of *CDKN1A* and *DDB2* targets and immunoblot of p53 protein levels at 6 and 24 h posttreatment in C4‐2 (p53 WT) and DU145 (p53 mutant) cell lines. Immunoblot is representative of three independent replicates (*n* = 3) in C4‐2 and DU145. Error bars on qPCR graph represent standard deviation (SD) between (D, E) three independent experiments in C4‐2 (*n* = 3) and DU145 (*n* = 3). N, Niraparib; O, Olaparib; Veh, Vehicle. Student's *T*‐test was used to determine statistical significance from vehicle. *P < 0.05, **P < 0.01, ns, not significant. For C4‐2, Olaparib *CDKN1A P*‐value: 0.0032, Niraparib *CDKN1A P*‐value: 0.0015. For C4‐2, Olaparib *DDB2 P*‐value: 0.0014, Niraparib *DDB2 P*‐value: 0.0164. For DU145, Olaparib *CDKN1A P*‐value: 0.5558, Niraparib *CDKN1A P*‐value: 0.0020. For DU145, Olaparib *DDB2 P*‐value: 0.8777, Niraparib *DDB2 P*‐value: 0.7795.

After assessing the overlap between all five PARPi, the more focused studies were aimed at detailed comparisons between the transcriptional effects of Olaparib and Niraparib. Olaparib and Niraparib have clinical relevance in Pca, as they are approved for use and in active clinical trials for the disease. As mentioned above, Olaparib and Niraparib treatment also impacted the largest number of genes (Fig. 3A). In Olaparib‐treated cells, 1913 of 2721 (70%) genes were uniquely impacted. For Niraparib‐treated cells, 85 out of 893 (9.3%) genes were uniquely impacted. Olaparib and Niraparib treatment commonly impacted 808 genes.

Pathway analysis (Websgetsalt) was performed on the differentially expressed genes (DEGs) across these two PARPi treatments [53]. Several p53‐related pathways were significantly enriched in response to both PARPi treatments. Importantly, genes transcriptionally regulated by p53 (TP53 Regulates Metabolic Genes, TP53 Regulates Transcription of DNA Repair Genes, and Transcriptional Regulation by TP53) were impacted by PARP inhibition, including genes involved in DNA repair and metabolic pathways (Fig. 3B).

Due to the significant enrichment of p53 pathways in response to Olaparib and Niraparib treatment, the DEGs between the two inhibitors were assessed. Most of the p53 regulated genes were upregulated in response to both Olaparib‐ and Niraparib‐treated cells (Fig. 3C). To validate these findings, two well‐known p53 target genes that were impacted by PARPi, *CDKN1A* and *DDB2*, were assessed by qRT‐PCR. At the mRNA level, *CDKN1A* and *DDB2* are significantly upregulated in cells treated with either Olaparib or Niraparib when compared to the vehicle control (Fig. 3D). In addition to validating the Olaparib and Niraparib results from the transcriptome‐wide assessment, the impact of the other three PARPi on these targets was assessed in C4‐2 using qRT‐PCR (Fig. S6A). Rucaparib and Talazoparib modestly, but significantly, upregulated both *CDKN1A* and *DDB2*. Combined, these data indicate that PARPi generally elicits activation of key p53 target genes associated with cell cycle arrest and DNA damage repair in an HRR‐competent model of CRPC (Contrasting with the RNA‐seq results, *CDKN1A and DDB2* expression in Veliparib conditions showed no change by qRT‐PCR.).

The p53 locus in C4‐2 cells is wild‐type [54]. In order to assess whether the upregulation of p53‐related genes is a generalizable effect of PARP inhibition treatment in models expressing WT p53, qRT‐PCR was performed on two other wild‐type expressing Pca cell lines (LNCap and 22RV1) (Fig. S6B,C). It was hypothesized that in these wild‐type p53 models, PARPi would similarly induce the expression of these p53 target genes (*CDKN1A* and *DDB2*), thus validating the RNA sequencing finding as a generalizable result in a p53 wild‐type setting. In LNCaP cells, Veliparib, Rucaparib, Olaparib, Niraparib, and Talazoparib treatment significantly upregulates *CDKN1A* expression via qRT‐PCR (Fig. S6B). Niraparib, Rucaparib, and Talazoparib significantly upregulate *DDB2* expression via qRT‐PCR. LNCaP cells treated with Veliparib and Olaparib demonstrated a slight increase in *DDB2*, which was not statistically significant. In 22RV1, another p53 WT model, *CDKN1A* is significantly upregulated by Rucaparib, Niraparib, and Talazoparib (Fig. S6C). *DDB2* expression is also slightly upregulated by Rucaparib, Niraparib, and Talazoparib in this model, again without statistical significance. These data indicate that in p53 WT Pca cell model systems, p53 target genes are activated in response to PARPi.

It was hypothesized that the upregulation of p53 target genes might be the result of p53 stabilization after PARPi treatment in p53 WT models. RNA‐seq experiments performed on PARPi‐treated C4‐2 cells were performed and validated at 24 h posttreatment; however, studies suggest that p53 is stabilized in response to stress at earlier time points [55]. Therefore, it was hypothesized that PARP inhibition might stabilize p53 at earlier time points, thus causing the increase in p53 target gene expression at the 24 h time point. At 6 h, in C4‐2 cells, p53 was increased 2–3‐fold in response to both Olaparib and Niraparib (Fig. 3D). By 24 h, p53 levels had returned to normal in both Olaparib‐ and Niraparib‐treated cells. At both time points, camptothecin (CPT) was used as a positive control, as it is known to stabilize p53 [56]. These data indicate that p53 protein stabilization is associated with the upregulation of p53 target gene expression in response to Olaparib and Niraparib treatments.

As mentioned, C4‐2 cells express wild‐type (WT) p53. Therefore, it was hypothesized that p53 mutation may impact how genes respond to Olaparib and Niraparib treatment. DU145 cells are p53 mutants due to missense mutations in the DNA binding domain [54]. In DU145, Olaparib and Talazoparib treatment do not impact *CDKN1A* expression (Fig. S6D). Furthermore, *CDKN1A* expression is downregulated in response to Niraparib, Veliparib, and Rucaparib (Fig. S6D). *DDB2* expression was not significantly impacted by any of the PARPi when compared to vehicle control (Fig. S6E).

Collectively, these results indicate that in response to PARPi, p53‐competent models upregulated p53‐related target genes. In C4‐2, target gene upregulation correlates with p53 stabilization. In contrast, PARPi treatment in the p53 mutant cell line DU145 did not result in the same pattern of p53 target gene upregulation. In the C4‐2 model, it was noteworthy that the increase in expression of p53 target genes was correlated with the IC50 values, in that Niraparib (IC50: 0.9 μm) upregulated the target genes (*CDKN1A* and *DDB2*) more robustly than Olaparib (IC50: 1.28 μm).

### 3.5 Manipulation of p53 expression impacts PARPi response in HRR‐competent Pca model systems

**Fig. 4:**
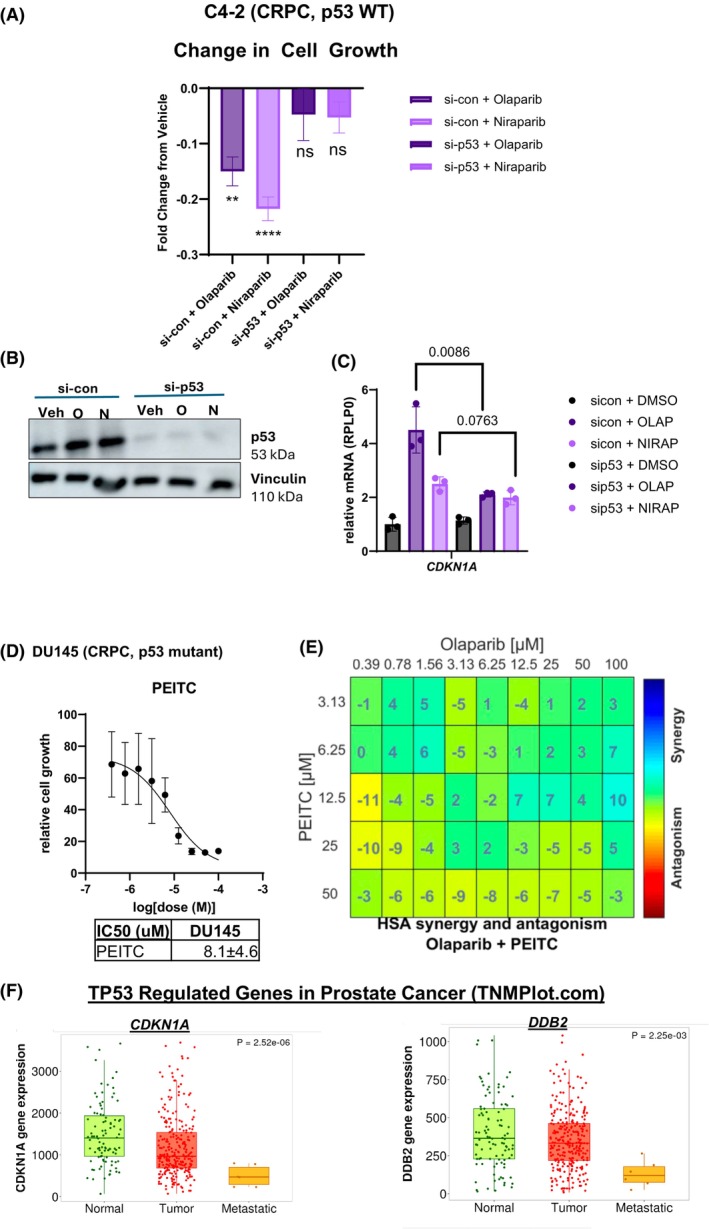
Genetic and therapeutic manipulation of p53 alters PARPi response. (A) Relative fold change 92 h post PARP inhibitor treatment in regular growth conditions with non‐targeting si‐RNA or si‐TP53. *P*‐values are in comparison to si‐control vehicle control or si‐p53 vehicle control. *P*‐values for relevant comparisons are reported from Student's *T*‐test. Error bars represent SEM between four independent experiments. **P < 0.001, ****P < 0.00001, ns, not significant. Si‐con + Olaparib *P*‐value: 0.0012, si‐con + Niraparib *P*‐value: < 0.0001, si‐p53 + Olaparib *P*‐value: 0.3492, si‐p53 + Niraparib *P*‐value: 0.1109. (B) Immunoblot validation of C4‐2 si‐TP53 knockdown representative of three independent replicates (*n* = 3). N, Niraparib; O, Olaparib; Veh, Vehicle. (C) RT‐qPCR of *CDKN1A* expression in non‐targeting si‐RNA versus si‐TP53 conditions after treatment with vehicle control or PARP inhibitor. Si‐control + DMSO and si‐TP53 + DMSO were set to 1. DMSO, dimethyl sulfoxide (vehicle); NIRAP, Niraparib; OLAP, Olaparib. Student's *T*‐test was used to generate *P*‐values. Error bars represent SD (standard deviation) between three independent experiments. (D) DU145 IC50 value for PEITC. PEITC, Phenethyl isothiocyanate. Quantified with dsDNA Quantiflor 1. Error bars represent standard deviation between three independent experiments. (E) Synergy chart generated with CompSci after treatment with Olaparib and/or PEITC. Synergy experiments were based on results from nine independent experiments. (F) *CDKN1A* and *DDB2* expression across normal, tumoral, and metastatic prostate tissue from TNMplot.com. Normal (*n* = 106), Tumor (*n* = 283), Metastatic (*n* = 6).

Considering Olaparib and Niraparib enriched p53‐related pathways in a p53 competent model system, assessment of p53 manipulation on PARPi response was prioritized. The C4‐2 WT p53 cell line was transiently depleted of p53. Following treatment with PARPi in C4‐2 WT p53 cells, growth was significantly decreased as compared to vehicle control, as expected (Fig. 4A). C4‐2 with decreased p53, however, did not decrease cell growth significantly in response to PARPi treatment as compared to its vehicle control (Fig. 4A). P53 depletion was validated via immunoblot (Fig. 4B). *CDKN1A* expression was increased in response to PARPi treatment in p53 WT C4‐2, as shown previously (Fig. 4C). PARPi treatment in C4‐2 with p53 knockdown did not upregulate *CDKN1A* expression as robustly after PARPi treatment. This indicates that depleting p53 attenuated *CDKN1A* expression following PARPi treatment. WT p53 C4‐2 treated with Olaparib showed a statistically higher upregulation in *CDKN1A* as compared to si‐TP53 C4‐2 treated with Olaparib. There was a trend towards higher upregulation of *CDKN1A* in Niraparib‐treated WT‐p53 C4‐2 as compared to si‐TP53, but it was not statistically significant. Collectively, these results indicate that p53 activity has a role in impacting the responsiveness and p53 pathway activation of cells to PARP inhibition in HRR‐competent Pca model systems.

In addition to genetic depletion of p53's impact on PARPi response, the therapeutic activation of p53 on PARPi response was assessed. Phenethyl isothiocyanate (PEITC) can selectively restore p53 function in DU145 cells, which carry two p53 mutations in p53 [57, 58]. The IC50 value for PEITC was 8.1 μm, similar to a previously published value (Fig. 4D) [57]. After determining this IC50 value, synergism analyses were conducted to assess potential beneficial dose combinations of synergistic value in a p53 mutant model system. Results revealed that there were areas of synergism between Olaparib and PEITC in DU145 (Fig. 4E). PEITC can be utilized in future studies to assess the impact of p53 therapeutic reactivation on PARPi response. Current results suggest that there is synergistic potential with PEITC and Olaparib, suggesting that p53 reactivation in a p53 mutant cell line may influence Olaparib responsiveness.

### 3.6 Potential of p53 status as a marker for response to PARPi in Pca

Due to PARPi treatment increasing *CDK1NA* and *DDB2* at the mRNA level in Pca, assessment of the expression of these genes in publicly available Pca datasets was performed using TNM plot. *CDKN1A* and *DDB2* expression is lower in more advanced Pca (Fig. 4F). Between normal and tumor tissue, there is a decrease in both *CDKN1A* and *DDB2*. Between normal and metastatic disease, there is a statistically significant decrease in both *CDKN1A* and *DDB2* levels. Lastly, between tumor and metastasis, there is a statistically significant drop in *CDKN1A* and *DDB2* levels. The associated p53 mutational status could not be matched with *CDKN1A* and *DDB2 expression* for the cohorts due to the nature of the TNMPlot data. However, two candidate datasets from TNMPlot, TCGA and SU2C, were analyzed to assess p53 mutational status impact on the selected p53 target genes. In general, within both datasets, lower expression of *CDKN1A* and *DDB2* were correlated with mutated *TP53* (Fig. S7A,B). These results indicate that in PCa, these genes often become dysregulated, typically downregulated between normal, tumor, and metastatic disease. Previous studies indicated that there may be differences between the expression of p53‐ and PARP1‐regulated DNA damage pathways between AA and EA patients [59, 60].

Considering p53 status was important to PARPi responsiveness, it was hypothesized that p53 status and target gene expression may impact PARPi responsiveness in AA patients as compared to EA patients. When compared in primary and metastatic datasets, there were no substantial differences between AA and EA p53 mutational burden. Although there were no differences in p53 mutational burden, it was hypothesized that there may be differences in p53 target gene expression between AA and EA samples. To assess the potential difference between AA and EA expression of p53 target genes in PCa samples, cbioportal datasets for primary (TCGA) and metastatic (SU2C) PCa were assessed and categorized by race [61]. In primary disease, there is no difference between *CDKN1A* or *DDB2* expression between racial groups (Fig. S7E,F). In the metastatic dataset, *CDKN1A* and *DDB2* levels trend higher in AA samples compared to EA patient samples (Fig. S7E,F).

Collectively, this study assessed PARP1 expression and activity in AA and EA samples, indicating that there may be differences in expression, which could lead to differences in treatment response. The studies also highlight the importance of p53 pathway upregulation in response to PARPi treatment in an HRR‐competent, CRPC model system.

## 4 Discussion

PCa remains the second leading cause of cancer‐related death in men, with Black men being disproportionately impacted by the disease [1]. This study identified differences between: (a) AA and EA PCa tumor samples, in terms of PARP1 and PAR expression and (b) PARPi response *in vitro* between an AA‐derived and EA‐derived model systems. Furthermore, different PARPi can differentially impact the transcriptome in HRR‐competent CRPC. Key findings from this study indicate that: (a) Clinically relevant PARPi elicit distinct antitumor activity in HRR‐competent PCa cells, (b) these PARPi elicit both overlapping and distinct changes in gene expression, (c) p53‐related pathways are elevated in response to Olaparib and Niraparib treatment in the subset of PCa models harboring wild‐type p53, (d) genetic and therapeutic manipulation of p53 can impact PARPi responsiveness and (e) there may be value in assessing p53 status as a marker for response to PARPi in PCa.

A previous study found that PARP1 protein expression was not significantly different between AA and EA samples [6]. The focus of the study was to assess the generalizable differences between DNA lesions expressed by AA and EA, not necessarily sensitivity to PARP inhibitor treatment. To date, there are no other studies assessing PARP1 and PAR levels using race as a comparator in PCa related studies. In the current study, PARP1 total protein and PAR levels were assessed, and samples were separated by disease aggressiveness (Gleason numbers). The interest behind assessment of PARylation in this study is driven by the fact that PARP inhibitors work by decreasing elevated PARP1 activity (PARylation). Previous work has indicated that PAR expression increases as a result of disease progression in PCa, making PARP inhibitors a viable treatment option for the disease [8, 43, 62]. Because our results suggest that in AA tissue samples, there is not an increase in PARylation between disease states, AA may not benefit as much from PARPi as compared to EA. Our EA samples (although a smaller amount than our AA sample set) showed the same trend as previously published data, indicating that this difference between AA and EA may help increase personalized treatment options for patients from different racial backgrounds.

Despite the transcriptomic differences between inhibitors, through downstream analysis, the unbiased approach utilized here is the first to demonstrate that p53 activity is increased in response to both Olaparib and Niraparib in a p53 WT, HRR‐competent PCa cell line model (C4‐2). This observation was further validated in two separate, wild‐type p53 cell models, thus generalizing the fact that in a p53 WT HRR‐competent setting, p53 pathway activity is upregulated (LNCaP, 22RV1). In contrast, in the context of a p53‐incompetent, HRR‐incompetent cell line DU145, p53 activity and expression were not increased in response to either PARPi. Overall, these data elucidate PARPi function in HRR‐competent contexts and broaden our understanding of how this signaling axis could be therapeutically exploited by PARPi, as either a single agent or in combination with other therapeutic options. The data presented in this study also provide rationale for future studies aimed at gaining a better understanding of the potential racial differences with respect to PARPi response.

The effect of PARPi in HRR‐competent models has not been extensively explored until recently. In fact, HRR‐competent models are often used in these studies to demonstrate the decreased sensitivity of these models to PARPi response. In an HRR‐competent colorectal cancer model, Olaparib increased p53 target gene expression and stabilization [47]. These studies were done in colorectal and breast cancer model systems and interrogated p53 activation at doses of PARPi up to 50 μm. Although multiple doses of Olaparib were tested (from 0 to 50 μm), no IC50 values were established for Olaparib in the cell lines. As a result, we lack a clear understanding of how PARPi increases p53 activity at a physiologically relevant dose. In contrast to the studies mentioned, the current study assessed the impact of five clinically relevant PARPi across a panel of different PCa cell lines with different genetic backgrounds, HRR status, and hormone therapy sensitivities, all at biologically relevant doses. Thus, the current study adds value to our understanding in HRR‐competent settings by demonstrating that p53‐related pathways are upregulated.

RNA sequencing in an earlier PCa study revealed that in both C4‐2B and LNCaP (p53 wild‐type cell lines) elevated expression of p53 target genes *CDKN1A*, *DDB2*, *BTG2*, and *NDRG1* occurred upon treatment with the PARPi Olaparib [46]. In this analysis, the p53 pathway was significantly enriched among 271 of the genes impacted by Olaparib treatment in LNCaP and C4‐2. Although both the current study and the study mentioned herein focused on a panel of PCa cells, the current study focused on the impact of each of five clinically relevant PARPi and the unique and distinct changes in response to these inhibitors. Additionally, the current study compared the impact of these five PARPi in the same cell model system (C4‐2). No study to date has examined the impact of the five different clinical PARPi tested for transcriptome changes. These analyses emphasize that different PARPi differentially impact the transcriptome. All four genes (*DDB2*, *UQCRQ*, *CDKN1A*, and *RPS4X*) that were upregulated by all five PARPi in this study have been associated with the p53 pathway [48, 63, 64, 65, 66].

Although novel, this study has limitations. The tissue samples within the study were obtained from PCa patients; however, there were no matched adjacent non‐neoplastic samples for this cohort. Future studies should focus on understanding the difference in PARP1 expression and activity in normal versus tumor tissue samples between EA and AA patient samples to see if there is also a difference in baseline expression. The tissue samples used herein had self‐reported race data. While valuable, future studies will have to be performed with genetically confirmed racially diverse model systems to understand the potential racial differences that may be driving these differences in PARPi expression and drug responsiveness, including p53 expression and status as it relates to PARPi response in PCa. The majority of the cell lines used in this study are of largely European ancestry (LNCaP and C4‐2 exhibit 100% EA ancestry; 22RV1 and DU145 are 99% EA ancestry [67, 68]). One AA‐derived PCa cell line, MDA‐PCa 2B, was used as a comparator to these models, which have 80% AA ancestry. Future studies are planned to examine the impact of PARPi in another racially diverse PCa model, such as RC‐77 T/E, which also exhibits 80% African ancestry [68]. Recent efforts have also been focused on creating new AA‐derived PCa cell lines, which could allow more robust testing of these preliminary observed race‐associated differences implied within the clinical samples in our study [69, 70]. An assessment of how PARPi differentially impacts the transcriptome, based on ancestral differences, could be of significant value. Clinical trial data suggest that PARPi may have differential benefit in the AA community/population in PCa [71]. In a PCa clinical trial, the hazard ratio for AA benefit to the combination therapy was not reported due to the small sample size. However, EA patients saw a clear benefit from the combination, with a hazard ratio of 0.62. Although not powered for race, this study indicates that there may be differential benefits for AA and EA that should be further explored to see if this treatment combination is or is not beneficial for AA patients.

Importantly, using RNAi to induce an HRR defect, our findings show that PARPi may work through different mechanisms of action in the presence or absence of p53. The paucity of cell models in general, and specifically harboring HRR defects, is a pervasive barrier in the PCa field. Future studies assessing the impact of PARPi on endogenous HRR‐incompetent cell models versus HRR‐competent cell models, will rely on the development of the HRR‐incompetent models. It will be essential for future studies to assess the mechanistic impact of PARPi in this HRR‐competent setting, in order to better understand how and why PARPi response may elicit upregulation of p53.

## 5 Conclusions

Taken together, these studies reveal that there are differences between AA and EA PARP expression in PCa and that PARPi impacts p53 upregulation in HRR/p53 WT model systems. Racially diverse patient tissue sample analysis, unbiased RNA sequencing, and biological assessment and manipulation of p53 expression reveal novel insights about the PARPi response in PCa model systems. In addition to these key findings, this study broadens our understanding of how the different PARPi impact the PCa transcriptome, an important step toward our ultimate goal of assigning individual patients to specific PARPi regimens.

## Conflict of interest

The authors declare no conflict of interest.

## Author contributions

MLC carried out substantial contributions to the conception, design, acquisition, analysis, interpretation of the data, and drafted the work. AAS, MJS, and SBM provided substantial contributions to conception, design, data analysis, critically reviewed the work for intellectual content, and gave final approval of the version to be published. JV‐G, SMB, ST, LJ, RM, GL, TFA, ESA, KR, JD, SK, DP, HS, and TM made substantial contributions to the acquisition, analysis, and interpretation of the data herein. JV‐G, ST, LJ, RM, HS, TM, and DP provided substantial data acquisition and analysis/interpretation of the data. GL, TFA, and ESA contributed to substantial data acquisition and analysis for Incucyte experiments. KR, JD, and SK provided MDA‐PCa 2B dose response curves. SBM conducted all bioinformatics analysis. NA and CS provided pathology scoring. LGG and WKK provided TMA slides.

## Disclaimers

The contents of this publication are the sole responsibility of the author(s) and do not necessarily reflect the views, opinions, or policies of Uniformed Services University of the Health Sciences (USUHS), The Henry M. Jackson Foundation for the Advancement of Military Medicine, Inc., the Department of Defense (DoD), the Departments of the Army, Navy, or Air Force. Mention of trade names, commercial products, or organizations does not imply endorsement by the U.S. Government.

## Supporting information


**Fig. S1.** PARP1 and PAR differences based on race in AA and EA model systems.
**Fig. S2.** PARPi dose response curves and impact on PARP1 expression in other PCa model systems.
**Fig. S3.** PARPi impact PARylation but not PARP1 expression in HRR‐competent models.
**Fig. S4.** Clinical PARPi elicit both overlapping and distinct changes in gene expression.
**Fig. S5.** Clinical PARPi pathways impacted by PARPi response.
**Fig. S6.** p53‐related pathways are enriched in p53 competent PARPi‐treated cell lines.
**Fig. S7.** P53 mutational status and associated CDKN1A and DDB2 expression data.
Table S1.

## Data Availability

The RNA sequencing (RNA‐seq) data generated in this study is available in Gene Expression Omnibus (GEO) at GSE289437. Additional data are presented as S1.
