# Supplementary material for: PARP inhibitors elicit distinct transcriptional programs in homologous recombination competent castration‐resistant prostate cancer
Source: Mol Oncol. 2025 Sep 7;20(2):369–88. doi: 10.1002/1878-0261.70098 (PMC12936415; doi:10.1002/1878-0261.70098)
Supplement: Supplementary file 1 — Fig. S1. PARP1 and PAR differences based on race in AA and EA model systems. Fig. S2. PARPi dose response curves and impact on PARP1 expression in other PCa model systems. Fig. S3. PARPi impact PARylation but not PARP1 expression in HRR‐competent models. Fig. S4. Clinical PARPi elicit both overlapping and distinct changes in gene expression. Fig. S5. Clinical PARPi pathways impacted by PARPi response. Fig. S6. p53‐related pathways are enriched in p53 competent PARPi‐treated cell lines. Fig. S7. P53 mutational status and associated CDKN1A and DDB2 expression data. Table S1. [file MOL2-20-369-s001.zip › Full_supplemental_figure_legends_SM.docx]

**Supplemental figure legends:**

**Supplemental Figure 1. PARP1 and PAR differences based on race in AA and EA model systems.** PAR and PARP1 scores were generated via the equation: (intensity * 1) + (percentage *2). The scale of 0 through 9 represents the calculated immunohistochemistry expression score for the samples within the cohort. PAR and PARP1 scores were compared between Gleason 6 and Gleason 7 CRPC patient samples. Results for **A,** **C and D** are reported as the percent of total patients with the pathologist score 0 through 9. **A.** PARP1 (n=46) and PAR (n=47) percentage of expression scoring in the entire cohort (AA samples + EA samples). **B.** PARP1 and PAR percentage of expression scores for Gleason 6 PCa TMA samples in EA (PARP1 n = 9; PAR n = 8) v. AA patients (PARP1 n = 38; PAR n = 38). **C.** PARP1 expression score in EA (n=9) v. AA (n=38) samples. **D.** PAR expression score in EA (n=8) v. AA (n=38) samples. **E** AA = African American, EA = European American.**.** PARPi IC50 in an AA derived PCa cell line, MDA-PCa-2B (AR+, CRPC). Cells were treated with vehicle control (DMSO) or PARPi (Veliparib, Rucaparib, Olaparib or Niraparib) for 96h. MTT assay was performed to determine IC50 values. Standard deviation represents three independent replicates. IC50 values in the table underneath the graphs represented are IC50 ± SD.

**Supplemental Figure 2. PARPi dose response curves and impact on PARP1 expression in other PCa model systems. A-D**. Efficacy of five different PARP inhibitors in C4-2 (AR+, CRPC), LNCap (AR+; HTS PCa), 22RV1 (AR+, AR-sv+; CRPC), and DU-145 (AR-, CRPC) models (respectively). Cells were treated with one of the 5 PARPi for 72h. Crystal Violet assay was performed to develop IC50 curves. IC50 values were reported in µM. Standard deviation represents three independent replicates. IC50 values in the tables to the right of the graphs are the IC50 ± SD. PARPi = PARP inhibitor, SD= standard deviation.

**Supplemental Figure 3. PARPi impact PARylation but not PARP1 expression in HRR competent models.** Quantification of blots in Figure 1F-G, **A.** C4-2 (AR+, CRPC) and **B.** DU145 (AR-, CRPC). Graphs and standard deviation (SD) are representative of three, independent replicates. PARPi IC50 curves and immunoblots of PARP1 and PAR expression in **C.** LNCaP (AR+, HTS) and **D.** 22RV1 (AR-SV, CRPC). V= Veliparib, O= Olaparib, R=Rucaparib, N = Niraparib, T= Talazoparib. Immunoblots presented are representative of three, independent replicates (n=3). Error bars on graphs are representative of standard deviation (SD) of three, independent replicates. Immunoblots are representative of three, independent replicates. **E.** Table of IC50 curves calculated via dsDNA one assay across all cell lines with IC50 ± SD of three, independent replicates. **F.** *BRCA2* transient knockdown in C4-2 cells. Cells were transfected with si-control (si-con) or si-BRCA2. Cells were treated with vehicle control (DMSO) or a PARPi (Talazoparib). Results were from 96h time point after analysis in the Incucyte. SEM represents results from three independent replicates. Statistics generated with a t-test. Veh = Vehicle, T= Talazoparib.

**Supplemental Figure 4. Clinical PARPi elicit both overlapping and distinct changes in gene expression. A.** Heatmap of differentially expressed genes after each PARPi treatment relative to vehicle control (DMSO).

**Supplemental Figure 5. Clinical PARPi pathways impacted by PARPi response. A.** Hallmark and KEGG Pathways Enriched after PARPi (Veliparib, Niraparib, Olaparib, Rucaparib, and/or Talazoparib) treatment. 28 pathways impacted metabolism, 12 impacted cell cycle, 12 impacted neurobiology, 10 impacted immune response, 9 impacted carcinogenesis, 5 impacted DNA repair, 3 impacted cell death, 2 impacted angiogenesis, 2 impacted transporters, 2 impacted cell adhesion, 2 impacted hormones, 1 impacted cell differentiation, and 1 impacted growth factor signaling.

**Supplemental Figure 6. p53 related pathways are enriched in p53 competent PARPi treated cell lines.** Relative expression of *CDKN1A* and *DDB2* across PARPi treatments. **A.** C4-2, **B.** LNCaP, **C.** 22RV1, and **D.** DU145. Immunoblot quantification of p53 levels at 6h and 24h post-treatment in **A.** C4-2 and **D.** DU145. Immunoblots shown are representative of three, independent replicates. Student’s T-tests were performed between PARPi and vehicle control to generate p-values. * p<0.05, ** p<0.001, *** p<0.0001. Error bars represent standard deviation between three, independent replicates. CRPC = castrate resistant prostate cancer.

**Supplemental Figure 7. p53 mutational status and associated CDKN1A and DDB2 expression data** for **A.** TGCA PanCan Atlas (n=493) and **B.** SUC2 (n=266), 2019 datasets. Datasets accessed April 21, 2025. Z-score comparison of C-D. p53 mutational status separated by European American and African American for **C.** TGCA PanCan Atlas and **D.** SU2C, 2019 datasets. **E.** CDKN1A mRNA and **F.** DDB2 mRNA from primary PCa (TGCA, Firehose, Legacy) versus metastatic PCa (SU2C, 2019) samples from cbioportal.com. Samples were categorized by patient race for TGCA (EA n= 147 v. AA n =7) and SU2C (EA n= 156 v. AA n = 18). Student’s T-test was performed to determine signifigance values. PCa = prostate cancer, mCRPC = metastatic castrate reistant prostate cancer. TCGA = The Cancer Gemone Atlas, SU2C = Stand up to Cancer.
