# Supplementary figures and images for: PARP inhibitors elicit distinct transcriptional programs in homologous recombination competent castration‐resistant prostate cancer
Source: Mol Oncol. 2025 Sep 7;20(2):369–88. doi: 10.1002/1878-0261.70098 (PMC12936415; doi:10.1002/1878-0261.70098)

Supplemental Figure 4

A.

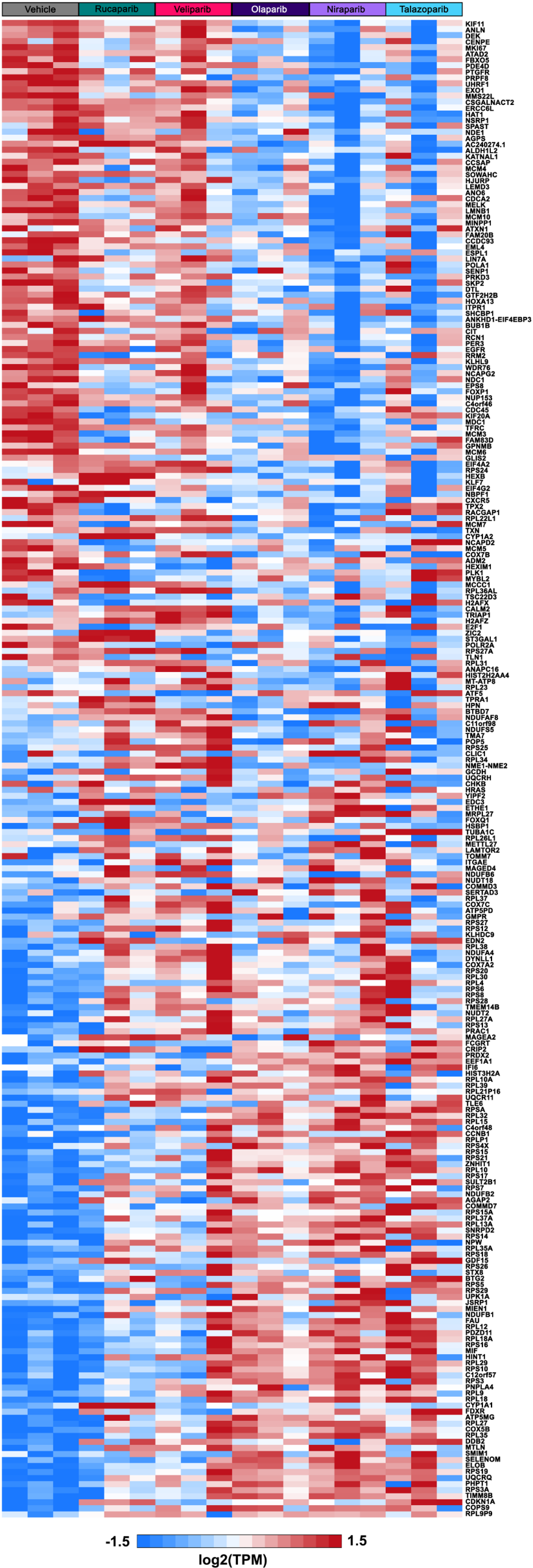

-1.5 1.5  
log2(TPM)

Supplement: Supplementary file 1 — Fig. S1. PARP1 and PAR differences based on race in AA and EA model systems. Fig. S2. PARPi dose response curves and impact on PARP1 expression in other PCa model systems. Fig. S3. PARPi impact PARylation but not PARP1 expression in HRR‐competent models. Fig. S4. Clinical PARPi elicit both overlapping and distinct changes in gene expression. Fig. S5. Clinical PARPi pathways impacted by PARPi response. Fig. S6. p53‐related pathways are enriched in p53 competent PARPi‐treated cell lines. Fig. S7. P53 mutational status and associated CDKN1A and DDB2 expression data. Table S1. [file MOL2-20-369-s001.zip › Figure S4.pdf]

**Supplemental Figure 5**

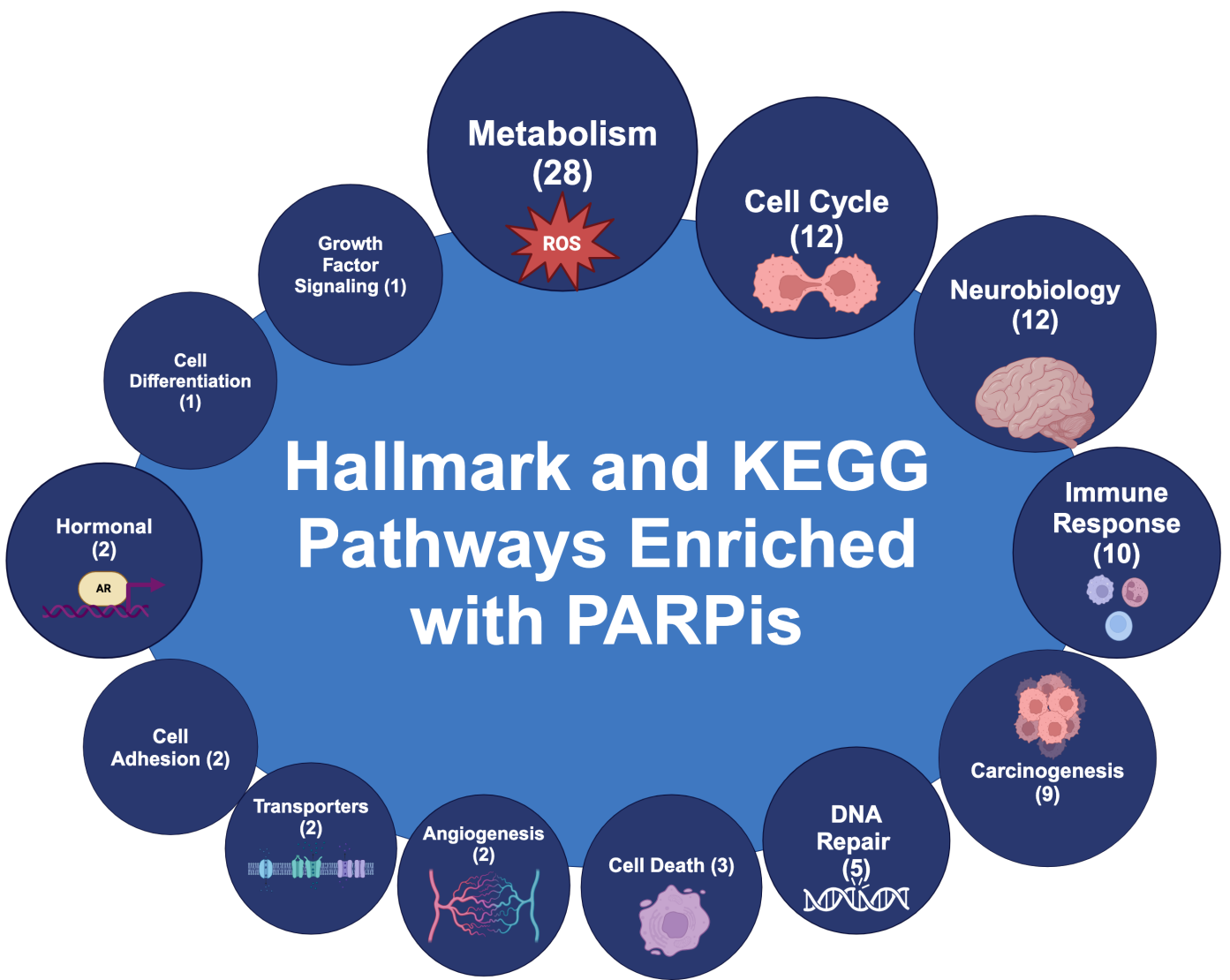

Supplement: Supplementary file 1 — Fig. S1. PARP1 and PAR differences based on race in AA and EA model systems. Fig. S2. PARPi dose response curves and impact on PARP1 expression in other PCa model systems. Fig. S3. PARPi impact PARylation but not PARP1 expression in HRR‐competent models. Fig. S4. Clinical PARPi elicit both overlapping and distinct changes in gene expression. Fig. S5. Clinical PARPi pathways impacted by PARPi response. Fig. S6. p53‐related pathways are enriched in p53 competent PARPi‐treated cell lines. Fig. S7. P53 mutational status and associated CDKN1A and DDB2 expression data. Table S1. [file MOL2-20-369-s001.zip › Figure S5.pdf]
